# Modifiable Risk Factors and Preventative Strategies for Severe Retinopathy of Prematurity

**DOI:** 10.3390/life13051075

**Published:** 2023-04-24

**Authors:** Minali Prasad, Ellen C. Ingolfsland, Stephen P. Christiansen

**Affiliations:** 1 Boston University Chobanian & Avedisian School of Medicine, Boston, MA 02118, USA; 2 Department of Pediatrics, Division of Neonatology, University of Minnesota, Minneapolis, MN 55455, USA; 3 Departments of Ophthalmology and Pediatrics, Boston University Chobanian & Avedisian School of Medicine, Boston Medical Center, Boston, MA 02118, USA

**Keywords:** severe retinopathy of prematurity, risk factor, protective strategies, oxygen, maternal chorioamnionitis, sepsis, blood transfusion, weight gain

## Abstract

Severe ROP is characterized by the development of retinal fibrovascular proliferation that may progress to retinal detachment. The purpose of this report is to review five of the most common and well-studied perinatal and neonatal modifiable risk factors for the development of severe ROP. Hyperoxemia, hypoxia, and associated prolonged respiratory support are linked to the development of severe ROP. While there is a well-established association between clinical maternal chorioamnionitis and severe ROP, there is greater variability between histologic chorioamnionitis and severe ROP. Neonatal sepsis, including both bacterial and fungal subtypes, are independent predictors of severe ROP in preterm infants. Although there is limited evidence related to platelet transfusions, the risk of severe ROP increases with the number and volume of red blood cell transfusions. Poor postnatal weight gain within the first six weeks of life is also strongly tied to the development of severe ROP. We also discuss preventative strategies that may reduce the risk of severe ROP. Limited evidence-based studies exist regarding the protective effects of caffeine, human milk, and vitamins A and E.

## 1. Introduction

Retinopathy of prematurity (ROP) is among the leading causes of blindness worldwide in children who are born preterm [1,2]. With advances in neonatal intensive care units and resuscitation efforts, more preterm babies survive and are at risk for developing ROP [3,4,5]. ROP is characterized by abnormal retinal blood vessel development in infants with low birth weight and low gestational age [6]. In modern cohorts, it affects 26.5–43% of infants screened for ROP in the United States, with 6–7% receiving treatment by laser or intravitreal anti-VEGF agents [7,8]. The disease burden is greater in developing regions, including India, China, Southeast Asia, and South America [4]. Compared to the US and other industrialized nations where ROP has a higher incidence in premature infants, babies from low- and middle-income countries, including those in Eastern Europe and Latin America, present with ROP at older ages and higher birthweights because of high mortality rates, which are secondary to variability in resources and differences in the availability of oxygen blending and saturation monitoring [9,10]. There is also considerable variation in the screening criteria between countries [11].

Currently, a two-phase hypothesis of the pathophysiology of ROP is accepted [5]. Phase 1, which typically occurs between 22 to 30 weeks post-conception, is characterized by retinal vascular attenuation. Compared to the naturally hypoxic intrauterine environment, the relatively hyperoxic environment experienced after birth suppresses the production and release of pro-angiogenic factors and growth factors, including vascular endothelial growth factor (VEGF) and insulin growth factor-1 (IGF-1), which results in an arrest of normal vascular development [6,12,13]. Phase 2, typically occurring between 31–36 weeks post-conception, is characterized by rapid vessel growth and neovascularization. The metabolically active retina at this post-conceptual age induces local hypoxia and increases VEGF and other pro-angiogenic factors with subsequent abnormal vessel proliferation. These vessels can form fibrous scars connecting the retina to the vitreous humor, and retraction of the scar tissue can lead to tractional retinal detachment and blindness [6,12,13].

ROP is diagnosed by means of indirect ophthalmoscopy, fluorescein angiography, or spectral domain optical coherence tomography [14]. The diagnosis and classification of ROP are based on location, severity, and vascular characteristics in the posterior pole, according to the International Committee for Classification of Retinopathy of Prematurity guidelines [15,16,17,18,19]. Three circumferential zones with increasing radii are centered on the optic nerve, which parallels the progression of retinal vascular development (from the optic nerve to the ora serrata) [17]. The severity of ROP ranges from a demarcation line between the avascular and vascular retina to a three-dimensional ridge, leading to extraretinal fibrovascular proliferation and, finally, to partial or total retinal detachment [17]. The retinal detachment is further characterized by its extent in clock-hour sectors [16]. Patients may also present with venous dilatation and arteriolar tortuosity. The presence of both vascular changes indicates “plus” disease and may be accompanied by iris vascular engorgement, pupillary rigidity, and vitreous haze [17]. “Pre-plus” disease is also characterized by vessel dilatation and tortuosity; however, the severity of these vascular changes is insufficient to diagnose “plus” disease [15]. Aggressive posterior ROP is characterized by rapidly progressive neovascularization with arteriovenous shunting and non-progressive plus disease [18].

The treatment of ROP has been guided by multiple landmark clinical trials over the past five decades, including the Cryotherapy for Retinopathy of Prematurity Cooperative Group (CRYO-ROP), the Early Treatment for Retinopathy of Prematurity Cooperative Group (ETROP), and the Bevacizumab Eliminates the Angiogenic Threat of Retinopathy of Prematurity (BEATROP) trials [20,21,22]. The preferred treatment for stage 3 ROP with plus disease in Zone I has transitioned from cryotherapy to argon and diode laser therapy or anti-VEGF therapy. In the BEATROP trial, intravitreal bevacizumab was found to be a superior treatment for stage 3+ retinopathy in zone I compared to laser therapy. Furthermore, unlike bevacizumab therapy, laser therapy impedes angiogenesis in the peripheral retina [20].

In the present work, we review the existing literature to highlight four preventative strategies and five modifiable perinatal and neonatal risk factors for the development of severe ROP, including oxygen exposure and associated respiratory support, maternal chorioamnionitis, red blood cell transfusions, sepsis, and poor weight gain. Notably, we exclude gestational age and birth weight as factors, as these are two of the most well-established predictive factors for severe ROP but are difficult to modify [23]. We define severe ROP as stage 3 ROP or higher, which clinically manifests as fibrovascular proliferation (stage 3) to retinal detachment (stages 4 and 5) [10]. While validated ranking tools do not exist to compare risk factors, Wu et al. reported the weighted importance of various clinical characteristics based on weight ratios from their deep learning system, and their rankings support the importance of the factors we have selected for further review [24].

## 2. Perinatal and Neonatal Risk Factors

### 2.1. Oxygen Exposure and Associated Respiratory Support

Hyperoxemia, hypoxemia, and substantial fluctuations in oxygen saturation are all correlated with the development of severe ROP. After birth, preterm infants are often resuscitated with supplemental oxygen therapy to increase arterial oxygen saturation. Previous studies found that a PaO_2_ greater than 80 mmHg increased the incidence of severe ROP by an odds ratio of 3:1 during the second through fourth weeks of life [25]. In a retrospective study, infants with severe ROP were exposed to a higher fraction of inspired oxygen (FiO_2_) and more frequent FiO_2_ titrations compared to infants without ROP. The Extremely Low Gestational Age Newborn (ELGAN) study reported an increased risk of ROP in zone 1 and plus disease with a PaO_2_ ≥ 100 mmHg or PCO_2_ ≤ 50 mmHg within the first three days of life [26]. Additionally, infants with severe ROP experienced iatrogenic hyperoxemia more frequently than those without ROP [27]. Hypoxemia is also pathogenic. Srivatsa et. al. found that extremely low birth weight infants who developed severe ROP experienced more hypoxemic episodes and spent more time in the severe hypoxemic range (oxygen saturation values below 80%) compared to infants without ROP [26]. Additionally, when oxygen therapy is discontinued, the resulting fluctuations in oxygen tension may lead to ROP development due to increased angiogenic factor production secondary to hypoxia of the underdeveloped and avascular retina [13,28,29]. Hypoxia-sensitive angiogenic factors, including VEGF and KDR/Flk-1 (VEGFR-2), mediate the pathologic vasoproliferation seen in severe ROP [28,30].

Newborn animal models have been used to mimic these oxygen fluctuations and study oxygen-induced retinopathy (OIR). Penn et al. developed a rat model such that animals were exposed to alternating hyperoxia (50% FiO_2_) and hypoxia (10% FiO_2_) every 24 h for 2 weeks to recreate the transcutaneous oxygen levels of human infants who develop severe ROP [31]. Penn et al. found that rats exposed to 50%/10% cycles experienced greater retinal vasculature retardation compared to those exposed to 80%/40% cycles. Other studies using the 50/10 OIR model found an increased avascular retinal area compared to pups raised in room air [32]. Rat models found that hyperoxic fluctuations were more likely to stunt retinal vascular development and produce more severe ROP compared to hypoxic fluctuations [33].

Both hyperoxia and hypoxia can lead to reactive oxygen species (ROS) generation, which is involved in the pathophysiology of ROP [34]. Hyperoxia increases superoxide levels and lipid peroxidation, while hypoxia upregulates oxidative enzyme activity [34]. Furthermore, infants have low antioxidant activity to defend against oxidative stress [35]. Increased activity of nicotinamide adenine dinucleotide phosphate (NADPH) oxidase, endothelial nitric oxide synthase, and gene products expressed after the stabilization of hypoxia-inducible factors mediates Phase 1 of ROP by producing ROS that lead to delayed physiologic retinal vascular development and an avascular retina [34,35]. The intravitreal neovascularization seen in Phase 2 of ROP is mediated in part by supplemental oxygen increasing signaling transducer and activator of transcription 3 (STAT3) activity [34]. Other biomarkers of oxidative stress in ROP include oxidized phenylalanine, plasma 8-hydroxy 2-deoxyguanosine, and serum malondialdehyde [35,36].

The development of oxygen-induced ROP in human newborns has been studied in several randomized controlled clinical trials to determine the appropriate oxygen levels needed to lower the risk of ROP, including severe ROP [29]. In the Surfactant, Positive Airway Pressure, Pulse Oximetry Randomized Trial (SUPPORT), preterm infants were randomized to either immediate intubation and surfactant administration or continuous positive airway pressure (CPAP) [37]. They were also randomly assigned to oxygen saturation levels at either 85–89% or 91–95% [37]. There was a lower incidence of severe ROP reported among infants maintained at the lower oxygen saturation range [37]. However, both the SUPPORT and the Benefits of Oxygen Saturation Targeting Study II (BOOST-II) reported increased mortality with the lower oxygen saturation range compared to higher oxygen saturation ranges [5,38]. The American Academy of Pediatrics committee on fetuses and newborns recommended higher target oxygen saturation levels of 90–95% in infants with extremely low birth weights to avoid increased infant mortality, despite the increased risk of ROP from hyperoxia [39].

The Supplemental Therapeutic Oxygen for Prethreshold Retinopathy of Prematurity (STOP-ROP) trial found that there was no difference in the development of threshold ROP between preterm infants with prethreshold ROP in at least one eye when they were maintained at either conventional oxygen levels (89–94% SaO_2_) or supplemental oxygen levels (96–99%) [40]. Results from the STOP-ROP trial also revealed that maintaining supplemental oxygen levels in infants without plus disease was protective against the progression to threshold ROP, compared to conventional oxygen levels [40]. However, infants maintained at supplemental oxygen levels were more likely to develop pneumonia and exacerbations of chronic lung disease, as well as to require oxygen, diuretics, or hospitalization at three months of age [40]. Overall, the optimal oxygen saturation levels to minimize severe ROP risk, as well as mortality, have yet to be determined. Raghuveer et al. suggested titrating oxygen saturation levels such that the infant is maintained under gradually increasing oxygen saturation levels as postmenstrual age increases to avoid early hyperoxia and later hypoxia [5].

Both independent of and cumulatively with oxygen exposure, positive pressure ventilation is an important modifiable risk factor for severe ROP [41,42,43,44,45]. The duration of mechanical ventilation is a risk factor for ROP development [46,47], and several studies have confirmed prolonged mechanical ventilation as an independent risk factor for ROP [48,49,50,51,52,53]. Similarly, it has been established that long-term use of a CPAP is predictive of treatment-requiring or severe ROP [41,42,43,44,45]. There were no significant differences in the incidence of ROP between infants treated with bi-level positive airway pressure or CPAP [54]. There was also no significant difference in the incidence of ROP among preterm infants maintained on bubble nasal CPAP or conventional nasal CPAP [55].

Preterm infants are often treated with steroids to treat inflammation related to bronchopulmonary dysplasia (BPD). The early administration of postnatal corticosteroids within the first seven days of life has been shown to decrease rates of ROP, including severe ROP, among survivors [56]. In contrast, the late administration (after the first seven days of life) of postnatal corticosteroids increased overall rates of severe ROP [57].

### 2.2. Maternal Chorioamnionitis

Maternal infection has been linked to preterm labor and delivery, as well as postnatal complications including periventricular leukomalacia, BPD, and cerebral palsy [58]. Chorioamnionitis is a maternal intra-amniotic infection characterized histologically by neutrophilic infiltration in the amnionic membrane, chorionic decidua, umbilical cord, or the chorionic plate [59,60,61]. It is diagnosed clinically by maternal fever in combination with maternal leukocytosis, purulent cervical discharge, or fetal tachycardia [59,62]. The most common organisms causing chorioamnionitis include *Ureaplasma urealyticum*, *Mycoplasma hominis*, *Gardnerella vaginallis*, bacteroides, and Group B streptococcus [59].

Antenatal inflammation plays a role in the pathogenesis of severe ROP [63]. Several studies have reported the association of clinical maternal chorioamnionitis with the development of any stage of ROP, including severe ROP, in preterm infants [60,64,65,66]. Premature infants screening positive for chorioamnionitis have a higher likelihood of developing aggressive posterior ROP compared to infants screening negative [67]. Infants with zone I ROP are more likely to screen positive for chorioamnionitis compared to infants with zone II ROP [67]. Although histologic chorioamnionitis (HCA) is classified by the extent and site of inflammation, severe ROP has been found to be significantly associated with all types of HCA [61,66,68,69]. Compared to placentas with vasculopathy, placentas with HCA have a higher risk of developing ROP [70]. An elevated maternal white blood cell count is also an independent risk factor for ROP [66].

However, the association between HCA and ROP is variable [71], with some studies reporting no significant association between the two [72,73]. Chen et al. reported that while the presence of placental bacteria or inflammation individually does not independently increase the risk of severe ROP in a preterm infant, the presence of both is associated with ROP in zone I [71]. On the contrary, the progression of acute histologic chorioamnionitis in infants without fetal growth restriction was found to be a protective factor against ROP in one study [74].

### 2.3. Sepsis

Postnatal infection is strongly associated with ROP. Neonatal sepsis is characterized by a bacterial or fungal infection within the first three months of life, with early-onset sepsis defined as sepsis within the first 72 h of life and late-onset sepsis defined as sepsis after the first 72 h of life [75,76]. The mechanisms by which sepsis promotes severe ROP are multifaceted. Sepsis leads to low retinal perfusion and ischemia. Sepsis causes white blood cells to form intravascular microthrombi, leading to obstruction and increased permeability in the retinal vasculature [75]. Oxidative stress causes vascular proliferation and necrosis, secondary to increased VEGF-2 and lipid peroxidation products, respectively [75]. Inflammatory cytokines such as interleukin-1β and transforming growth factor-β increase hypoxia-inducible factor-1α, which contributes to ROP [33,34,75].

Several studies have found an increased risk of the development of ROP, including severe ROP, among preterm infants with neonatal sepsis [77,78,79,80,81,82,83,84,85,86,87,88]. In a meta-analysis of 16 studies investigating the association between septicemia and ROP, Wang, et al. confirmed the existence of a significantly increased risk of any stage of ROP (odds ratio of 1.57, 95% CI = 1.31–1.89) and over twice the risk of severe ROP among preterm infants with sepsis (odds ratio of 2.33 95% CI = 1.21 to 4.51). Late sepsis (including both bacterial and fungal sources) was also found to be associated with ROP (odds ratio of 2.2, 95% CI = 1.5–3.2). Both early (odds ratio of 9.9, 95% CI = 1.2–83.4) and late bacterial sepsis (odds ratio of 2.0, 95% CI = 1.3–3.3) were also found to be associated with the development of ROP.

Additionally, neonatal fungal infection is independently associated with threshold ROP in very low birth weight (1000–1500 g) infants [89]. Mittal et al. reported that Candida sepsis is an independent predictor of severe ROP. Candida albicans is known to cause increased prostaglandin production by endothelial cells, leading to increased endothelial permeability and decreased neutrophil adherence [90]. Systemic fungal infections may affect the developing retinal vasculature by increasing the production of proinflammatory cytokines that mediate angiogenesis, leading to severe ROP [91]. Compared to C. albicans sepsis, Candida parapsilosis sepsis is linked to a higher incidence of ROP [92].

Necrotizing enterocolitis (NEC) is a gastrointestinal inflammatory or ischemic disease found in premature neonates and is classically characterized by pneumatosis intestinalis and portal venous gas upon abdominal radiograph [93]. There is an increased risk of ROP and severe ROP in neonates with medical or surgical NEC compared to infants without NEC [53,84,94,95]. Furthermore, there is a higher risk of ROP, including severe ROP, with early-onset surgical NEC (occurring between 8–28 days of life) compared to later-onset surgical NEC (occurring later than 28 days of life) [94].

### 2.4. Blood Transfusions and Erythropoietin

Preterm infants often receive transfusions for severe anemia due to their immature hematopoietic systems and the need for frequent phlebotomy to monitor their critical statuses [96]. Adverse reactions include infections, hemodynamic complications, electrolyte imbalances, and necrotizing enterocolitis [97]. Blood products contain hemoglobin A, which has a lower affinity for oxygen and greater dissociation from oxygen compared to fetal hemoglobin or hemoglobin F [98,99]. This may predispose infants to retinal vascular injury from exposure to hyperoxia and oxygen free radicals [98]. Lower hemoglobin F levels after transfusions given during inpatient admission were found to be associated with ROP development [100]. The Cord Blood Transfusion in Preterm study was a proof-of-concept work reporting that preterm neonates receiving cord red blood cell transfusions had higher hemoglobin F levels compared to neonates given adult red blood cell transfusions. Of the 23 neonates included in this study, 8 developed ROP (stage I (n = 1), stage II (n = 5), and stage III (n = 2)). Both of the neonates who developed stage III ROP had received a single adult red cell blood cell transfusion which was not followed by cord red blood cell transfusions. The neonates with stage I or stage II ROP received cord red blood cell transfusions alone (Stage I) or both cord and adult red blood cell transfusions (4/5 Stage II cases) [101]. The BORN (umBilical blOod to tRansfuse preterm Neonates) is an ongoing multicenter randomized clinical trial investigating severe ROP development among neonates receiving allogeneic cord red blood cell transfusions [102]. Blood transfusions increase serum iron levels, which may increase ROS production and lipid peroxidation, further contributing to oxidative stress [103]. Inder et al. reported that elevated iron, transferrin, and ferritin levels from transfusions within the first week of life were associated with an increased risk of ROP development. Transfusions have also been suggested to induce a recipient inflammatory response, which may or may not be related to the duration of red blood cell storage prior to transfusion [104]. The duration and severity of the anemia preceding transfusion have also been shown to be risk factors for ROP development [105,106].

The reported association between the frequency or volume of red blood cell transfusions and the development of ROP is variable and inconsistent [107,108,109,110,111,112], although the majority of studies suggest an increased risk of severe ROP with an increased number [81,107,113,114,115,116,117,118] or volume [119,120] of transfusions in preterm infants [86,87,88,121,122,123,124]. The timing of the transfusion also appears to have an influence, with the administration of products within the first week [125], 10 days [126], 30 days [127], or 60 days [125] of life increasing the likelihood of the development of ROP. Hesse et al. found a significantly increased incidence of ROP among infants receiving more than 45 mL/kg of blood compared to infants receiving a lower volume.

Erythropoietin (EPO) treatment is used to reduce the need for red blood cell transfusions in preterm or low birth weight infants [128,129,130]. However, as a hypoxia-sensitive pro-angiogenic factor, it may play a role in ROP development. Elevated endogenous serum EPO within the first two weeks of life has been linked to a significantly increased risk of developing ROP [129,131]. EPO administration, independent of the infant’s age, has been associated with severe ROP development, though there are conflicting data [128]. EPO may produce different effects on retinal vascularization depending on the phase of ROP [129]. Cochrane reviews have reported no increased risk of ROP with the early (before 8 days of life) initiation of erythropoietin. However, when compared to late administration (8–28 days of life), early EPO was associated with an increased risk of ROP [132,133]. The multi-site randomized Preterm Erythropoietin Neuroprotection Trial reported no difference in any ROP outcomes between infants receiving early high-dose EPO compared to those receiving a placebo [134].

Hengartner et al. also found that the administration of at least one platelet transfusion increased the risk of severe ROP. Similarly, Elgendy reported a significant association between infants receiving platelet transfusions and the development of ROP compared to infants not receiving platelet transfusions (22.3% vs. 19.2%, respectively, *p* < 0.001) [135]. Fresh frozen plasma (FFP) contains coagulation factors, platelets, and whole blood [136]. It also contains IGF-1 and IGF-1 binding protein-3, which have been shown to induce vascularization in retinopathy [137,138]. In a retrospective cohort study, Dani et al. reported that at least two FFP transfusions within the first two weeks of life have a protective effect against the development of ROP in preterm infants [137].

### 2.5. Poor Postnatal Weight Gain

Poor postnatal weight gain is a well-established, modifiable risk factor for ROP. The association between slow growth and ROP severity is best documented during the first weeks of life. Poor weight gain within the first six weeks of life has been shown to be associated with the development of severe ROP in several studies. Filho et al. reported that low weight gain within the first six weeks of life was an independent predictor of severe ROP among very low birth weight preterm neonates [139]. Wallace et al. similarly found an association between the poor rate of postnatal weight gain within the first six of weeks of life and the development of severe ROP [140]. Very low birth weight infants with severe ROP requiring laser treatment were found to have lower relative rates of weight gain in the second and fourth weeks of life compared to those with no ROP or mild ROP [141]. Anuk Ince et al. found that low weight gain in the fourth and sixth weeks of life was associated with severe ROP [142]. Kim et al. reported poor postnatal weight gain within the first two weeks of life as an independent risk factor for the development of severe ROP [143]. However, Ingolfsland et al. reported that slow growth from 32 to 37 weeks post-conception is also associated with ROP ≥ stage 2, suggesting that growth during both the avascular and neovascular phases of ROP development impacts the severity of ROP [144].

Several models have been developed and validated that expand on the traditional screening criteria for ROP to also include postnatal weight gain. The Weight, IGF, Neonatal Retinopathy of Prematurity (WINROP) study was the first to use postnatal weight gain in an algorithm that identified infants who had developed severe ROP [145]. The Postnatal Growth and Retinopathy of Prematurity Study (G-ROP) investigated the sensitivity of 6 screening criteria, including postnatal weight gain, within the first 39 days of life, in addition to birth weight and gestational age, in predicting type 1 and type 2 ROP. The G-ROP criteria, including postnatal weight gain, were more predictive than the screening criteria, which accounted only for birth weight and gestational age [7,146]. When applied to a cohort of premature infants with treatment-requiring ROP, the G-ROP model produced similar results [146]. The Colorado Retinopathy of Prematurity (CO-ROP) model required a weight gain of less than or equal to 650 g within the first month of life, in addition to low gestational age and birth weight, in order for ROP screening to be conducted [147]. The WINROP [148], G-ROP [146,149,150,151,152,153,154], and CO-ROP [155,156,157] algorithms, showing poor postnatal weight gain to be a sensitive and specific predictor of ROP severity, have been validated by a number of studies around the world.

## 3. Protective Strategies

In addition to efforts to minimize the effects of known risk factors, there exist some evidence-based, preventative therapies and strategies that may protect against severe ROP. As ROP is a multifactorial disease, using a combination of preventative strategies may be necessary to significantly reduce the ROP risk. First, maintaining infants on continuous pulse oximetry within target saturation parameters is an important strategy to reduce ROP. Continuous pulse oximetry allows providers to prevent lengthy episodes of hypoxia and hyperoxia, both of which have been implicated in ROP pathogenesis. Bullard et al. attributes the decreased incidence of all stages of ROP in part to continuous pulse oximetry [158,159]. Quality improvement initiatives to improve adherence to target saturation goals have improved ROP outcomes in several NICUs [160,161]. Gentle et al. found that an increase in time at the goal oxygen saturation (90–95%) from 48.7% to 57.6% was accompanied by a decrease in the rate of death or severe ROP, from 32.1% to 18.0%. Similarly, Chow et al. reported that an O_2_ management and monitoring program aiming to minimize the time in hyperoxia and hypoxia–hyperoxia fluctuations at a level 3 NICU reduced the incidence of stage III and stage IV ROP from 12.5% to 2.5%. Lau et al. used an algorithm to titrate the fraction of inspired oxygen such that each preterm infant was maintained within a target oxygen saturation range and reported a decreased risk of the development of severe ROP as well as the need for laser therapy [162].

Zhang et al. reported that caffeine therapy mitigated the pathological vaso-obliteration and angiogenesis mediated by hyperoxia and hypoxia, respectively, in oxygen-induced ROP [163]. They demonstrated that caffeine’s protective effect is mediated by A2AR-dependent and -independent mechanisms using murine knockout models [163]. However, Hussein et al. reported a significant association between greater cumulative caffeine dosage and the development of severe ROP requiring treatment [164]. Bhatt-Mehta et al. found no association between total caffeine exposure and the severity of ROP [165]. There was also no association between the duration of caffeine therapy and aggressive posterior ROP [166]. More research will be needed before caffeine treatment recommendations can be proposed for the management of infants at risk for severe ROP.

Human milk includes exogenous and endogenous antioxidants that are reported to be protective against ROP development [5,167,168]. Additionally, human milk contains IGF-1, which is a proangiogenic factor that promotes retinal vascularization [168]. Human milk is rich in long-chain polyunsaturated fatty acids (LCPUFA), and previous studies have reported significantly decreased development of severe ROP among preterm infants given a fish oil lipid emulsion containing ω-3 LCPUFA and or a soybean lipid emulsion containing ω-6 LCPUFA [169,170]. ω-3 and ω-6 LCPUFA mediate normal retinal vascular development and support retinal metabolism and neuronal development, respectively [171]. Decreased adiponectin, which is also found in human milk, is associated with the loss of ω-3 LCPUFA’s protective effect on vascularization, delayed vascularization, and imbalances in retinal lipid levels, which cumulatively increase the risk of ROP development in murine models [171]. As has been summarized by several systemic reviews, there is conflicting evidence regarding the role of feedings with any human milk compared to mother’s milk in preventing severe ROP. Zhou et al. reported an overall reduced incidence of severe ROP among infants exclusively fed human milk versus any formula, those mainly fed human milk versus mainly formula, and those exclusively fed human milk versus exclusive formula [172]. Schanler et al. found a significantly lower incidence of stage 3 ROP among infants fed with mother’s milk (5.6%) compared to infants fed with donor human milk (19%) or preterm formula (14%). However, the meta-analysis conducted by Raghuveer et al. showed a decreased odds ratio of 0.31 for ROP when infants were fed with any human milk (95% confidence interval 0.19–0.49, *p* < 0.001) [5,172,173].

Vitamin A and E were both found to counteract the pathophysiology underlying oxygen-induced retinopathy in animal models [174,175]. Vitamin A inhibits neovascularization by decreasing VEGF, and Vitamin E is an antioxidant that attenuates oxidative stress [5,174,175]. Vitamin E was found to reduce the risk of severe ROP, but not any ROP, among very low birth weight infants in a Cochrane review [176,177]. Vitamin A therapy has been shown to reduce the incidence of any ROP, including severe ROP, and lower Vitamin A levels in umbilical cord blood were associated with ROP development [36]. However, results from two recent meta-analyses are conflicting, with one showing no significant differences in the incidence of ROP between preterm infants receiving vitamin A compared to those not receiving the supplementation (OR 0.65, 95% CI 0.29–1.48) and the other showing a significantly lower odds ratio for any ROP among those receiving Vitamin A (OR 0.27, 95% CI 0.15–0.48) [178].

Other preventative strategies include topical ketorolac and propranolol. There is mixed evidence regarding the effectiveness of topical ketorolac. A preliminary report (n = 59) demonstrated a lower risk of progression to severe ROP among preterm newborns treated with topical ketorolac compared to oxygen therapy [179]. In contrast, another small randomized clinical trial (n = 47) concluded that there was no significant difference in the risk of severe ROP development between preterm newborns treated with topical ketorolac tromethamine and a placebo eye drop [180]. This variability warrants further investigation into the use of topical ketorolac with larger, multi-center randomized clinical trials. Β-adrenergic blockers such as propranolol decrease hypoxia-induced VEGF [181]. While propranolol 0.1% eye micro-drops did not affect the progression of ROP, propranolol 0.2% eye micro-drops reduced the progression to severe ROP, and were found to be more effective when administered during the proliferative phase. Systemic reviews and meta-analyses are needed to establish the effectiveness of both topical ketorolac and propranolol [182,183].

## 4. Conclusions and Future Directions

Our review outlines five common and modifiable risk factors underlying the development of severe retinopathy of prematurity and summarizes the evidence behind several protective strategies to decrease the incidence or severity of ROP. Supplemental oxygen exposure, hyperoxemia, hypoxemia, and positive pressure ventilation are strongly correlated with severe ROP, and minimizing exposure to these risk factors through the use of non-invasive ventilation and maintaining target oxygen saturations are important strategies for risk reduction. Maternal chorioamnionitis and postnatal sepsis involve inflammation, and independently increase the risk of severe ROP. While an increased number and volume of red blood cell transfusions are strongly tied to the development of severe ROP, there is a relative paucity of conclusive literature regarding the role of erythropoietin and platelet transfusions in the pathogenesis of severe ROP. Poor postnatal weight gain is also associated with severe ROP and may be used as part of other screening algorithms. Evidence-based preventative strategies include caffeine, human milk, and vitamins A and E, but these require validation in larger multi-center studies. Further research regarding the differences in risk factors for the development of severe ROP among high-, middle-, and low-income countries, as well as the most effective treatment methods in countries with variability in their access to resources, is warranted to lower the prevalence of severe ROP in countries with an elevated disease burden.

## Data Availability

Not applicable.
